# Decreased Erythrocyte CCS Content is a Biomarker of Copper Overload in Rats

**DOI:** 10.3390/ijms11072624

**Published:** 2010-07-02

**Authors:** Jesse Bertinato, Lindsey Sherrard, Louise J. Plouffe

**Affiliations:** Nutrition Research Division, Health Products and Food Branch, Health Canada, Sir Frederick G. Banting Research Centre, 251 Sir Frederick Banting Driveway, Ottawa, Ontario, Canada; E-Mails: lindsey.sherrard@hc-sc.gc.ca (L.S.); louise.j.plouffe@hc-sc.gc.ca (L.J.P.)

**Keywords:** copper overload, CCS, biomarker, rat, erythrocytes

## Abstract

Copper (Cu) is an essential trace metal that is toxic in excess. It is therefore important to be able to accurately assess Cu deficiency or overload. Cu chaperone for Cu/Zn superoxide dismutase (CCS) protein expression is elevated in tissues of Cu-deficient animals. Increased CCS content in erythrocytes is particularly sensitive to decreased Cu status. Given the lack of a non-invasive, sensitive and specific biomarker for the assessment of Cu excess, we investigated whether CCS expression in erythrocytes reflects Cu overload. Rats were fed diets containing normal or high levels of Cu for 13 weeks. Diets contained 6.3 ± 0.6 (Cu-N), 985 ± 14 (Cu-1000) or 1944 ± 19 (Cu-2000) mg Cu/kg diet. Rats showed a variable response to the high Cu diets. Some rats showed severe Cu toxicity, while other rats showed no visible signs of toxicity and grew normally. Also, some rats had high levels of Cu in liver, whereas others had liver Cu concentrations within the normal range. Erythrocyte CCS protein expression was 30% lower in Cu-2000 rats compared to Cu-N rats (P < 0.05). Notably, only rats that accumulated high levels of Cu in liver had lower erythrocyte CCS (47% reduction, P < 0.05) compared to rats fed normal levels of Cu. Together, these data indicate that decreased erythrocyte CCS content is associated with Cu overload in rats and should be evaluated further as a potential biomarker for assessing Cu excess in humans.

## 1. Introduction

Copper (Cu) is an essential trace metal that can cycle between reduced (Cu^+^) and oxidized (Cu^2+^) forms. This property allows Cu to act as a catalytic co-factor for several metalloenzymes involved in a number of biochemical processes including cellular respiration, connective tissue formation, neurotransmitter production, pigment synthesis, antioxidant defense and iron homeostasis [1]. The ease in which Cu can cycle between oxidation states makes Cu a potentially toxic metal if allowed to accumulate to high levels. “Free” Cu not tightly bound to proteins or other molecules can participate in Fenton-type reactions generating the toxic hydroxyl radical that can damage cellular components. Because Cu is both an essential and potentially toxic metal, cells have evolved transporters that regulate the uptake or elimination of Cu [2,3]. Also, Cu chaperones bind Cu in cells and deliver the metal to specific enzymes or subcellular compartments preventing the accumulation of “free” Cu [4].

Disruption of Cu-trafficking systems can lead to Cu deficiency or toxicity. Examples are the genetic disorders Menkes’ and Wilson’s disease. Menkes’ disease is caused by impaired activity of the Cu efflux transporter ATP7A which leads to a systemic Cu deficiency due to defective intestinal Cu uptake [5]. Wilson’s disease is the result of impaired activity of the Cu efflux transporter ATP7B [6,7]. Wilson’s disease results in Cu overload in liver and other tissues due to impaired biliary Cu excretion.

A nutritional Cu deficiency results in decreased activity of Cu-dependent enzymes and consequently decreased efficiency of a number of biochemical processes. A decrease in Cu status produces a number of biological changes and many of these changes have been proposed as biomarkers of Cu deficiency. These have been recently reviewed [8]. Importantly, fewer changes have been described in response to Cu overload. At present, an ideal biomarker for assessing Cu overload is lacking. Elevated Cu content in liver is currently regarded as the most reliable measure of Cu overload. However, this test requires a liver biopsy which is an invasive procedure. Thus, liver Cu measurement is unsuitable for routine screening and is only justified when Cu overload is suspected such as in patients with assumed Wilson’s disease. Furthermore, the uneven Cu distribution in liver may result in misdiagnosis of Cu overload with a single biopsy specimen [9–11].

Liver damage is a symptom of Cu toxicity. Alanine aminotransferase (ALT) and aspartate aminotransferase (AST) are elevated in serum when liver damage occurs and therefore can be used to assess Cu toxicity. However, increased ALT and AST levels are not specific for Cu toxicity and are only increased once tissue damage has occurred. Elevated 24 hour urine Cu content is also used to assess Cu overload, particularly in patients with Wilson’s disease [12]. Drawbacks of this test are that multiple urine samples must be collected and possible contamination of samples at collection. Urinary Cu may also be unsuitable for detection of more subtle increases in Cu load. Further, high urinary Cu may be unrelated to Cu excess [13]. Plasma or serum non-ceruloplasmin (Cp)-bound Cu is a promising biomarker for assessing Cu overload, although a convenient method for direct and accurate measurement of this Cu fraction is needed. Direct measurement of “free” Cu in serum or plasma ultrafiltrate (molecular weight cut-off of 30 kDa) by inductively coupled mass spectrometry revealed elevated levels in untreated patients with Wilson’s disease [14]. These data warrant further research on the specificity and sensitivity of this marker.

Given the lack of a sensitive and specific biomarker that can be measured with a simple, non-invasive test, research characterizing potentially better biomarkers of Cu overload is needed. Cu chaperone for Cu/Zn superoxide dismutase (CCS) is a homodimeric protein of ∼33 kDa subunits and functions to deliver Cu to the antioxidant enzyme Cu/Zn superoxide dismutase (SOD1) [15]. We have previously reported that CCS protein is increased in liver and erythrocytes of Cu-deficient rats [16]. Others have shown similar increases in CCS protein in tissues using different animal models of Cu deficiency [17–19]. Up-regulation of CCS under conditions of Cu deficiency was determined to result from decreased degradation of CCS protein by the 26S proteasome [20]. Further work showed that Cu binding to the CXC (C, cysteine; X, any amino acid) Cu-binding motif in the C-terminus of CCS decreases CCS stability and promotes proteasomal degradation [21].

CCS expression in erythrocytes is particularly sensitive to Cu deficiency. We have shown that erythrocyte CCS protein is a sensitive biomarker of mild Cu deficiency induced by moderately high intakes of zinc in rats [22]. Increased erythrocyte CCS expression was determined to be more sensitive to mild reductions in Cu status compared to plasma Cu concentration or Cp activity [22], the two most widely used markers for assessing Cu deficiency. A recent study has indicated that erythrocyte and liver CCS protein is increased in Cu-deficient calves and CCS may serve as a good biomarker of Cu deficiency in cattle [19]. Also, liver and erythrocyte CCS did not change in response to a vaccine-induced inflammatory response in beef heifers indicating that CCS expression was not affected by an inflammatory response unlike plasma Cu and Cp which are elevated under such conditions [19].

CCS is currently regarded as a promising biomarker for assessing Cu deficiency [8]. However, to our knowledge there are no reports describing regulation of CCS protein expression in response to Cu overload in animals. A human study showed that CCS and SOD1 mRNA were reduced in peripheral mononuclear cells of healthy adults with high serum Cp concentrations following supplementation with 10 mg Cu per day for 60 days [23]. Notably, however, similar reductions in CCS and SOD1 mRNA were not detected in subjects with low Cp concentrations. These data warrant further characterization of these markers. In this study, we measured CCS protein expression in erythrocytes of rats fed high Cu diets as a first step in determining the potential of erythrocyte CCS to serve as a biomarker for assessing Cu overload.

## 2. Results and Discussion

Rats were used to investigate whether erythrocyte CCS protein expression is altered in response to Cu overload. Although rats are tolerant to dietary Cu levels much greater than amounts needed to maintain adequate Cu status, dietary levels needed to induce increases in body Cu levels and toxicity have been established [24–27]. Rats were fed a diet containing normal amounts of Cu (Cu-N diet) or one of two diets containing high levels of Cu (diets Cu-1000 and Cu-2000) for 13 weeks. Diets contained 6.3 ± 0.6, 985 ± 14 or 1944 ± 19 mg Cu/kg diet by analysis (Table 1). For the entire duration of the study, all rats fed the Cu-1000 diet grew normally and did not show any visible signs of Cu toxicity. In contrast, 1 of the 12 rats fed the Cu-2000 diet was found dead after 6 weeks on the diet. An additional 4 rats in this diet group were euthanized due to weight loss, diarrhoea and dehydration after 7 (3 rats) and 10 (1 rat) weeks on the diet. Internal examination indicated damage to several tissues. Rats had a distended stomach and cecum, enlarged kidneys with a pale brown discolouration and rough surface and a moderately enlarged spleen. The liver had a pale brown discolouration.

Elevated levels of serum ALT and AST are indicative of damage to the liver or other tissues. Increased serum blood urea nitrogen (BUN) and creatinine levels are commonly used to diagnose impaired kidney function. Serum ALT levels were above the reference interval in 2 of the 4 euthanized rats, while serum AST, BUN and creatinine levels were elevated in all 4 of these rats (data not shown). Results of these biochemical tests are consistent with the morphological alterations indicative of Cu-induced damage to the liver, kidneys and other tissues. Haematological measurements indicated that these rats were also anaemic. They had low erythrocyte counts and Hb values (data not shown).

Rats have the ability to adapt to high dietary levels of Cu. Rats fed high levels of Cu (3,000–6,000 mg Cu/kg diet) showed an initial rapid rise in liver Cu concentration followed by a decline in liver Cu over several weeks [27]. Despite the severe Cu-induced toxicity observed in 5 rats fed the Cu-2000 diet, the remaining 7 rats in this diet group grew normally and did not show any adverse effects from the high Cu diet. Body weights of rats fed the Cu-N, Cu-1000 or Cu-2000 diets were similar after 13 weeks on the diets (Table 1). The resistance of some rats to severe Cu toxicity indicates that these rats adapted to the high Cu diet. The variable response of the rats may be explained, in part, by genetic heterogeneity of the Wistar rat strain which allowed some rats to better adapt to the high Cu diet.

Internal examination of rats fed the Cu-1000 or Cu-2000 diets that completed the study revealed no significant morphological changes to tissues. Most haematological parameters were similar for Cu-N, Cu-1000 or Cu-2000 rats (Table 2). However, MCHC values were lower and RDW values were higher for Cu-2000 rats compared to Cu-N rats indicating that the Cu-2000 diet induced a decrease in haemoglobin concentration in a packed volume of erythrocytes and larger variation in the size of erythrocytes.

Cp is a Cu-containing enzyme released into the circulation from the liver. Depressed Cp activity is a widely used marker for assessing Cu deficiency. Plasma Cp activity did not differ between rats fed normal or high Cu (Table 3). Serum ALT also did not differ in rats fed the different diets (Table 3). However, 1 rat fed the Cu-2000 diet had an ALT level above the reference interval. Serum AST was higher in Cu-2000 rats compared to Cu-N rats (Table 3) and 6 of 12 rats fed the Cu-1000 diet and 4 of 7 rats fed the Cu-2000 diet had AST levels above the reference interval. Since an elevation in AST is not specific for liver damage, we cannot say whether the increases in AST reflect liver damage or damage to other tissues. Notably, ALT which is considered a more specific enzyme for liver damage was within the reference interval for 18 of 19 rats fed the high Cu diets that completed the study. Serum BUN and creatinine levels were similar in Cu-N, Cu-1000 or Cu-2000 rats (Table 3) and levels were not elevated for any of the rats suggesting the absence of significantly impaired renal function in rats fed high Cu that completed the study.

**Figure 1.**
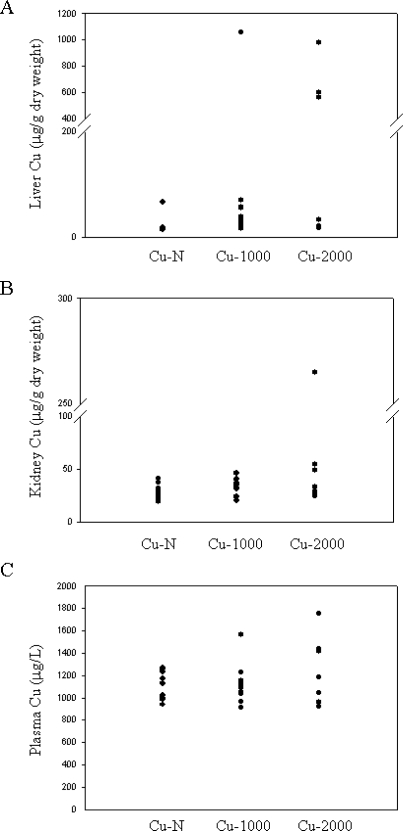
Cu concentrations in liver (**A**), kidney (**B**) and plasma (**C**) of rats fed diets with normal or high amounts of Cu. Each solid circle corresponds to 1 rat, n = 12, 12 and 7 for Cu-N, Cu-1000 and Cu-2000, respectively.

Liver Cu concentration for most rats fed high Cu were not markedly elevated (16–69 μg/g dry weight) (Figure 1A). In 4 rats, 1 rat fed the Cu-1000 diet and 3 rats fed the Cu-2000 diet, liver Cu concentration was markedly elevated (564–1058 μg/g dry weight) (Figure 1A). These levels are comparable to liver Cu concentrations seen in Wilson’s patients [11,28]. Despite high liver Cu, these rats did not show any visual signs of Cu toxicity. Only 1 rat (fed the Cu-2000 diet) had considerably elevated Cu in kidney (Figure 1B). This rat also had high liver Cu. Plasma Cu levels were not significantly different between Cu-N, Cu-1000 or Cu-2000 rats (P > 0.05) (Figure 1C). Notably, however, the highest plasma Cu concentrations were detected in the 4 rats that also had the highest liver Cu concentrations.

The variable response of rats to high dietary Cu was striking. Some rats showed normal Cu levels in tissues, while others accumulated high amounts of Cu. Furthermore, some rats showed severe Cu toxicity warranting euthanasia, whereas others accumulated high levels of Cu in tissues with little or no pathology. Although humans and rats differ in their sensitivity to Cu, these data merit research examining the interindividual variability of humans to Cu accumulation and toxicity in response to high Cu intakes. These data also stress the importance of assessing Cu status with a biomarker that reflects tissue Cu load rather than by Cu intake estimates.

**Figure 2.**
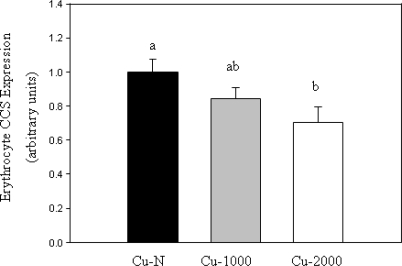
Relative CCS content in erythrocytes of rats fed the Cu-N, Cu-1000 or Cu-2000 diets. The mean of Cu-N rats was arbitrarily set to 1. CCS expression is expressed relative to GAPDH expression. Values are means ± SEM. Bars without a common letter differ, P < 0.05.

**Figure 3.**
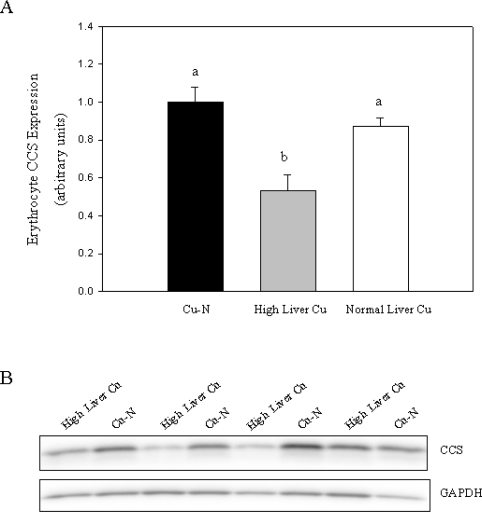
Relative CCS expression in erythrocytes of rats fed diets with normal amounts of Cu (Cu-N) or high levels of Cu displaying high (High Liver Cu) or normal (Normal Liver Cu) liver Cu concentrations. CCS expression in erythrocytes was quantified and expressed relative to GAPDH expression (**A**). The mean for Cu-N rats was arbitrarily set to 1. Values are means ± SEM, n = 7, 4 and 10 for Cu-N, High Liver Cu and Normal Liver Cu, respectively. Bars without a common letter differ, P < 0.05. Representative Western blot showing erythrocyte CCS expression in rats fed normal Cu (Cu-N) or rats fed high Cu and having high liver Cu concentrations (High Liver Cu) (**B**). CCS expression was detected with a CCS-specific antibody (top panel). The membrane was stripped and probed with an antibody against GAPDH (bottom panel).

CCS protein content in erythrocytes was measured by Western blot using a CCS-specific antibody. CCS expression was expressed relative to GAPDH content since erythrocyte GAPDH protein has been shown to be unaffected by changes in Cu status [17]. Erythrocyte CCS was reduced by 30% in Cu-2000 rats compared to Cu-N rats (Figure 2). CCS was not lower in Cu-1000 rats compared to Cu-N rats (Figure 2). Given that only some rats fed the high Cu diets accumulated high levels of Cu in liver, we chose to compare CCS expression in these rats with rats fed normal Cu or rats fed high Cu without marked accumulation of Cu in liver. Rats that accumulated high Cu in liver had lower CCS (47% decrease) compared to rats fed a normal Cu diet (Figure 3). Furthermore, rats with high liver Cu also had lower CCS compared to rats fed high Cu but did not show marked accumulation of Cu in liver (Figure 3A). Taken together, these results indicate that reduced CCS protein in erythrocytes is associated with increased body Cu load and not high dietary Cu intake per se.

In this study, we investigated the change in erythrocyte CCS in response to Cu overload only in male rats. It will therefore be important to test whether CCS responds in a similar manner in females. It should be noted, however, that CCS content has been shown to be elevated in tissues and erythrocytes of both male and female Cu-deficient rats and mice [17,18].

In cells, Cu binding to CCS decreases CCS stability and promotes its degradation by the 26S proteasome [20,21]. Decreased CCS protein in erythrocytes of Cu-loaded rats is consistent with the mechanism of CCS regulation by Cu. Given that mature erythrocytes are anucleated, regulation of CCS content in response to Cu overload may occur during erythropoiesis in maturing erythrocytes that have a nucleus and can efficiently support protein turnover. Under conditions of Cu overload, maturing erythrocytes may be exposed to higher levels of Cu. Higher cellular Cu concentrations would be expected to increase CCS degradation and consequently result in lower levels of CCS in mature erythrocytes. Notably, if CCS regulation by Cu occurs in maturing erythrocytes, a measurable decrease in CCS content in mature circulating erythrocytes would not be detected immediately following acute Cu overload. A detectable decrease in CCS would depend on the synthesis of a significant amount of new erythrocytes exposed to high Cu during maturation. Therefore, lower erythrocyte CCS content would be indicative of chronic Cu overload.

## 3. Experimental Section

### 3.1. Animals and Test Diets

Male 31-day-old Wistar rats (Charles River Canada, St. Constant, Canada) had free access to 1 of 3 test diets (n = 12/diet group) and demineralised drinking water. Diets were modified AIN-93G diets similar to diets described previously [16]. Cu was added to the diets as cupric carbonate. Diets differed only in Cu content. Cu concentrations in samples of each diet were determined by flame atomic absorption spectrophotometry (AAS) (Perkin-Elmer 5100 PC; Perkin Elmer Cetus Instruments, Norwalk, CT). After 13 weeks of feeding the diets, rats were killed by exsanguination while anesthetised with 3% isoflurane. Blood was collected from the abdominal aorta for biochemical tests and determination of haematological parameters. Blood was also collected in K_2_EDTA Trace Element tubes (Fisher Scientific, Ottawa, Canada) for isolation of erythrocytes and plasma. The liver and right kidney were extracted and snap frozen in liquid nitrogen and then stored at −80 °C until analysis. The Health Products and Food Branch Animal Care Committee of Health Canada approved the experimental protocol. Rats were treated in accordance with the guidelines of the Canadian Council on Animal Care.

### 3.2. Blood Fractionation

Blood samples were centrifuged at 1000 × g for 10 min at 4 °C. Plasma was collected and stored at −80°C until analysis. Erythrocytes were washed 3 times with cold isotonic 0.9% NaCl prior to freezing the erythrocyte pellet.

### 3.3. Biochemical and Haematological Measurements

Biochemical measurements in serum were determined using the Horiba ABX Pentra 400 clinical chemistry analyzer with ALT (A11A01627), AST (A11A01629), BUN (A11A01641) and creatinine (A11A01868) kits. Reference intervals were previously calculated using healthy 4–6 month-old Sprague Dawley, Fischer and Wistar rats fed a nutritionally complete rodent chow diet. Haematological parameters were determined using the Beckman Coulter AcT 5 Diff CP haematology analyzer. Plasma Cp activity was measured from its oxidase activity using *o*-dianisidine dihydrochloride as described [29].

### 3.4. Western Blotting

Erythrocytes were hypotonically lysed by adding 4 volumes of cold 10 mmol/L Tris pH 7.2 containing a protease inhibitor cocktail (Roche, Laval, Canada) and vortexing. Cellular debris was pelleted by centrifugation (1000 × g, 5 min, 4 °C) and the supernatant was retained for determination of Hb concentration using Drabkin’s reagent (Sigma, Oakville, Canada) and human haemoglobin as a reference standard (Sigma). Erythrocyte extracts (20 μg Hb) were separated over 8–16% Tris-Glycine gradient gels (Invitrogen, Burlington, Canada) under denaturing and reducing conditions. Gels were simultaneously electroblotted onto a single PVDF Immobilon-P transfer membrane (Millipore, Etobicoke, Canada). The membrane was blocked for 1 h at room temperature (RT) in TBS-Tween (20 mmol/L Tris, 500 mmol/L NaCl, 0.1% Tween 20 (v/v), pH 7.5) supplemented with 5% (wt/v) nonfat dry milk (Bio-Rad, Mississauga, Canada). Membranes were probed with an antibody against CCS (H-7, Santa Cruz Biotechnology, Santa Cruz, CA) at a final concentration of 0.4 mg/L overnight at 4 °C in TBS-Tween supplemented with 0.5% nonfat dry milk. After washing with TBS-Tween, membranes were incubated with an anti-mouse horseradish peroxidise-conjugated secondary antibody (Bio-Rad) at a 1:2500 dilution in TBS-Tween supplemented with 0.5% nonfat dry milk for 2 h at RT. Antibody-bound proteins were detected by enhanced chemiluminescence using SuperSignal® West Dura Extended Duration Substrate (Thermo Scientific, Rockford, IL). Membranes were stripped with stripping buffer (62.5 mmol/L Tris-HCl pH 6.8, 2% SDS (wt/v), 100 mmol/L 2-mercaptoethanol) for 30 min at 55 °C and re-probed with an antibody against glyceraldehyde-3-phosphate dehydrogenase (GAPDH) (MCA-1D4, Encor Biotech, Alachua, FL) at a 1:1000 dilution. Images were captured with a Chemi Genius^2^ Bio Imaging System (PerkinElmer, Woodbridge, Canada) and the intensities of the bands were quantified using Scion Image software (Scion Corporation, Frederick, MD).

### 3.5. Cu Determination in Tissues

Cu content in liver and kidney was determined by flame AAS after ashing the tissues as described [30]. Cu concentrations were presented per gram of dry tissue weight. Plasma Cu concentration was measured using a SIMAA 6000 graphite furnace AAS with Zeeman background correction (Perkin-Elmer Cetus Instruments, Shelton, CT). Plasma was diluted 1/20 in demineralised water prior to Cu determination. Cu concentrations in tissues and plasma were determined from a standard curve prepared using an NIST-certified reference standard.

### 3.6. Statistical Analyses

Data were analysed by one-way ANOVA and differences between means were determined by Tukey’s honestly significant difference (HSD) test or Unequal N HSD test. Data were reported as means ± SEM. Statistical significance was set at P < 0.05. Data were analysed using Statistica 8 software (StatSoft, Tulsa, OK).

## 4. Conclusions

This work is the first demonstration that CCS protein is down-regulated in response to Cu overload in animals. Since erythrocyte CCS content can be measured in a small blood sample, it is appealing as a potential biomarker for assessing Cu excess. Further research should examine CCS expression in erythrocytes of humans with Cu overload. Erythrocyte CCS content may prove useful as a biomarker for assessing Cu overload and as a diagnostic test for disorders of Cu overload such as Wilson’s disease.

## Figures and Tables

**Table 1.** Cu content in test diets and body weight of rats 1.

**Diet Group**	**Test Diets (mg Cu/kg diet) 2**	**Initial Body Weight (g) 3**	**Final Body weight (g) 3**
Cu-N	6.3 ± 0.6 ^a^	112 ± 2.9 ^a^	554 ± 6.5 ^a^
Cu-1000	985 ± 14 ^b^	109 ± 2.7 ^a^	571 ± 16 ^a^
Cu-2000	1944 ± 19 ^c^	114 ± 1.0 ^a^	603 ± 15 ^a,^^4^

1 Values are means ± SEM. Values in a column without a common letter differ, P < 0.05.

2 n = 3.

3 n = 12.

4 n = 7.

**Table 2.** Erythrocytes (ERCS), haemoglobin (Hb), haematocrit (HCT), mean corpuscular volume (MCV), mean corpuscular haemoglobin (MCH), mean corpuscular haemoglobin concentration (MCHC), red cell distribution width (RDW), platelet count (PLT), mean platelet volume (MPV) and white blood cells (WBC) of rats fed diets normal or high in Cu for 13 weeks 1.

**Diet Group (n)**	**ERCS (10^12^/L)**	**Hb (g/L)**	**HCT**	**MCV (fL)**	**MCH (pg)**	**MCHC (g/L)**	**RDW (%)**	**PLT (10^9^/L**	**MPV (fL)**	**WBC (10^9^/L)**
Cu-N (12)	7.8 ± 0.1 ^a^	138 ± 2^a^	0.40 ± 0.01^a^	51.5 ± 0.2^a^	17.8 ± 0.1^a^	344 ± 1^a^	12.2 ± 0.2^a^	714 ± 29^a^	6.6 ± 0.08^a^	3.9 ± 0.3^a^
Cu-1000 (12)	8.1 ± 0.1^a^	138 ± 1^a^	0.40 ± 0.00^a^	50.3 ± 0.4^a^	17.1 ± 0.2^a^	341 ± 1^a,b^	13.1 ± 0.2^a,b^	723 ± 36^a^	6.6 ± 0.08^a^	4.5 ± 0.4^a^
Cu-2000 (7)	8.0 ± 0.1^a^	134 ± 4^a^	0.40 ± 0.01^a^	49.3 ± 1.2^a^	16.7 ± 0.5^a^	338 ± 2^b^	13.7 ± 0.5^b^	777 ± 29^a^	6.6 ± 0.16^a^	4.9 ± 0.3^a^

1 Values are means ± SEM. Values in a column without a common letter differ, P < 0.05.

**Table 3.** Plasma Cp activity and serum ALT, AST, BUN and creatinine of rats fed diets normal or high in Cu for 13 weeks 1,2,3.

**Diet Group (n)**	**Cp (U/L)**	**ALT (U/L)**	**AST (U/L)**	**BUN (mg/dL)**	**Creatinine (μmol/L)**
Cu-N (12)	168 ± 7.0 ^a^ (140 - 208)	36 ± 5.1^a^ (23 - 87)	89 ± 5.1^a^ (62 - 113)	17 ± 0.8^a^ (13 - 23)	39 ± 1.7^a^ (30 - 50)
Cu-1000 (12)	163 ± 7.7^a^ (127 - 230)	50 ± 5.7^a^ (29 - 102)	156 ± 19^a,b^ (67 - 302)	17 ± 1.3^a^ (12 - 26)	37 ± 2.3^a^ (25 - 52)
Cu-2000 (7)	180 ± 10^a^ (141 - 222)	80 ± 25^a^ (25 - 202)	253 ± 68^b^ (87 - 532)	15 ± 1.1^a^ (12 - 20)	39 ± 2.4^a^ (29 - 48)

1 Values are means ± SEM. Values in a column without a common letter differ, P < 0.05.

2 The data range is indicated in parenthesis below each value.

3 Reference intervals: ALT (0-186 U/L); AST (0-156 U/L); BUN (12.62–26.09 mg/dL); creatinine (31.6–65.6 μmol/L).
